# The impacts of incentive policies on improving private investment for rural electrification in Nigeria – A geospatial study

**DOI:** 10.1016/j.heliyon.2024.e27440

**Published:** 2024-02-29

**Authors:** Munir Husein, Magda Moner-Girona, Giacomo Falchetta, Nicolò Stevanato, Fernando Fahl, Sandor Szabó

**Affiliations:** a Center for Clean Energy and Climate Change, Baze University, Abuja, Nigeria; b European Commission, Joint Research Centre (JRC), Ispra, Italy; c International Institute for Applied Systems Analysis, Laxenburg, Austria; d Euro-Mediterranean Center on Climate Change, Venice, Italy; e Department of Energy, Politecnico di Milano, Italy; f European Dynamics, Luxembourg

**Keywords:** Electricity access, Mini-grid, Incentives, Regulatory framework, Geospatial analysis, Nigeria

## Abstract

In Nigeria, 86 million people lack electricity access, the highest number worldwide, predominantly in rural areas. Despite government efforts, constrained budgets necessitate private investors, who, without adequate incentives, are hesitant to commit capital due to perceived high risks. This study identifies three existing incentive policies—concessionary loans, capital subsidy, and financing productive use equipment—aimed at promoting rural electrification in Nigeria. Employing geospatial and regulatory analyses, we evaluate their impact on electrification planning across 22,696 population clusters. While all incentives encourage mini-grids and stand-alone systems, results show varied impacts, predominantly favouring mini-grids. Under the baseline, grid extension is optimal for 66% of clusters, followed by mini-grids (27%) and stand-alone systems (7%). Concessionary loans boost mini-grid and Stand-Alone Systems shares by 10% and 5%, respectively. Capital subsidies increase the mini-grid share to 41%, surpassing concessional loans (37%). Financing productive equipment enhances Stand-Alone Systems and mini-grid shares to 15% and 43%. Incentives impact LCOE, CAPEX, and OPEX, with average LCOE reducing to 0.31 EUR/kWh (concessionary loans), 0.30 EUR/kWh (capital subsidy), and 0.27 EUR/kWh (financing productive use). Financing productive uses proves decisively more effective in lowering costs for mini-grids and stand-alone systems than loans or capital subsidies. The important policy implications of this study reinforce the need for tailored incentives for distinct electrification options.

## 1 Introduction

### 1.1 Background and motivation

The Sustainable Development Goal 7 (SDG 7) of the 2030 United Nations Agenda identifies affordable and clean energy as a key enabler in global initiatives to reduce poverty and improve human and societal well-being [1]. However, 745 million people are still living without access to electricity [2] in 2023, the majority in rural areas of Sub-Saharan Africa and Southern Asia. This is despite the numerous benefits of electricity access, which are well-documented [3]. These benefits include economic empowerment [4], health improvement [5,6], educational benefits [[7], [8], [9]], socio-economic change and welfare [10,11], and environmental and food production benefits [[12], [13], [14]].

Lack of electricity access is particularly problematic in Sub-Saharan Africa, where the access rate is 50%, meaning 598.8 million people still lack access to electricity in 2023 [2]. Even within Sub-Saharan Africa, the access rate is uneven between countries. Nigeria, Africa's most populous country with the largest economy, ranked first in the world by electricity access deficit, with 86 million people lacking access in 2021. In Nigeria, electricity access progress is slow and barely outpacing population growth [15,16]. This makes the country a crucial hotspot of intervention in pursuit of universal access by 2030.

The main technological options for electricity access include national grid extension, mini-grids, and stand-alone systems (SASs) [17,18]. Unlike SASs, grid extension and mini-grid allow productive uses of electricity as they can provide sufficient power for energy-intensive equipment. Traditionally, both mini-grids and SASs are developed by governments, but due to the constraints in the public budget, privately financed mini-grids and SASs are getting more attention [19]. However, private investors still perceive these investments as high-risk and low-return investments [20,21], constraining the expansion of the mini-grid and SASs market [22].

Indeed, policymakers in Nigeria have acknowledged the significance of mini-grids and SASs and the role of private investors in addressing energy poverty in the country, particularly in rural areas. Nigerian Electricity Regulatory Commission (NERC) has developed a Mini-grid Regulation [23], and the Nigerian Rural Electrification Agency (REA) recently partnered World Bank and African Development Bank to provide various incentives to the private sector to improve electricity access through mini-grid and SASs.

For private investors to commit capital, and for governments to provide incentives, a large-scale electrification planning is required. The planning is needed to determine the best technologies and electrification options to deploy in each community, as well as the government to understand the quantity and type of incentive required. For large-scale electrification planning, GIS-based models are increasingly applied [24], partly due to rising open data availability. These models consider local factors such as population density, existing and planned transmission networks and power plants, economic activities, tariffs for grid-based electricity, technology costs for mini-grid and off-grid systems, renewable energy potentials, and fuel costs, among others.

Examples of these GIS-based models applied at the continental level include [[25], [26], [27], [28], [29]]. A comparative study [30] has identified the SDG and multilayer data integration of the six most used geospatial planning tools at a continental level. Studies at the country level in Africa were conducted in Nigeria [31,32], Kenya [[33], [34], [35]], Burkina Faso and Côte d’Ivoire [36,37], Ghana [38,39], Mozambique [40], Ethiopia [41], Malawi [42] and Uganda [43], other studies are conducted at the regional level [44].

None of the studies mentioned above integrate the impact of incentive policies in electrification planning despite the existence of such policies in developing countries subsidizing mini-grids and SASs [45,46]. Also, another key element missing in all the previous studies is the subsidy given to the National Utilities. A survey of 39 national utilities in Africa indicates that they receive explicit or implicit subsidies of more than 40% of their connection costs, enabling them to sell electricity at prices that are 41%–80% less than the utilities’ unsubsidized LCOE [47,48] This is crucial since the studies are comparing the LCOE of all the electrification options, therefore, not accounting for the National Utility subsidy will result in a biased comparison.

While the number of country-scale electrification planning models and studies is increasing, the key novel contributions of this study are the following:

i) The study selects the three most significant national electrification incentive policies in Nigeria. The three incentives are assessed by the Off-PVGIS model which allows us to analyse and compare various types of electrification options under the different policies. We carry out this analysis from the private investors' perspective.
ii) We model population clusters (communities) at a 100 m spatial resolution, including the specific characteristics of each community: density of population, distance to the grid, and socio-economic conditions depending on the location of the cluster. The hourly load profile of each community is computed, based on latitude, socio-economic conditions, and climate area of the cluster. This leads to the possibility of matching load curves and variable renewable energy sources (VRES) availability curves at an hourly level, leading to an improved understanding of the role of each energy source in energy planning [49].
iii) We extend the capabilities of national-scale electrification models in two ways:
  a) We enhance the optimization process by performing a detailed hourly simulation of each of the three electrification options.
  b) The economics of the systems are simulated for the lifetime of the projects using the cash flow technique. The assessment is designed under the specific key parameters for Nigerian incentive policies and conditions—these results in a geospatial output with more accurate LCOE computation.

The remainder of the paper is organized as follows: Section 2 describes the methodology, including the model of distributed energy resources, the performance simulation model, and the economic simulation model. In Section 3, the datasets underlying the study are described and analysed. Section 4 presents the results of the impact of three incentive policies. Finally, the conclusion, policy implications, and areas for further studies are presented in Section 5.

## 2 Methodology

### 2.1 Model overview

**Fig. 1:**
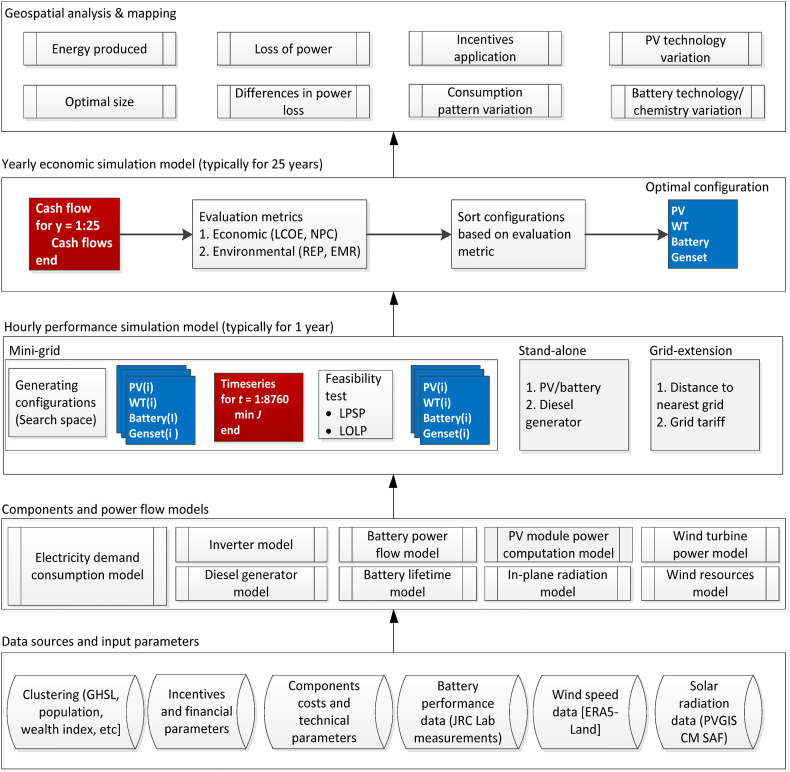
Flowchart diagram of the structure of the off-PVGIS model, data sources and input parameters.

The off-PVGIS model, as presented in this paper (schematised in Fig. 1), builds upon the model extensively detailed in Huld et al., 2017 [25,50], and has been applied in earlier studies by Refs. [35,[51], [52], [53]]. The foundational model has been strengthened and expanded using methodologies outlined in Refs. [54,55]. It is a large-scale electrification planning tool that uses: 1) Population-density data (GHSL) and geospatial properties for un-electrified clusters, 2) Estimated annual hourly electricity consumption of the clusters 3) Annual weather data (solar radiation, wind speed, temperature, etc.) with an hour resolution, 4) Technical and cost parameters of various electrification options. The objective of the model is to find an optimal electrification option that will meet the total electricity demand of each cluster. The possible electrification options are grid extension, mini-grid, and stand-alone system (SAS), which are summarized in Table 1. The optimal electrification option is determined by calculating the levelized cost of electricity (LCOE) of all the systems. The electrification system with the lowest LCOE is then selected as the optimal electrification option for that cluster.

**Table 1:** Electrification options considered.

Electrification option	Energy source
Grid extension	National grid
Mini-grid	Solar PV/BESS
Stand-alone system	Solar PV/BESS

In the model, the options for electrification through a mini-grid may include any configuration containing solar PV, wind turbines, small-hydro, batteries, and diesel or gas-powered generators. However, in this study, only solar PV back-up with batteries mini-grid is considered. In the case of SASs solar PV and battery systems are considered. The business model favours these systems over diesel generators for several reasons: they are generally almost always more economically viable, require less or no maintenance, and are more environmentally friendly. The grid extension is the average national grid LCOE and an additional LCOE of the transmission and distribution infrastructure.

The model of distributed energy resources (DERs) is presented, which includes the model of solar photovoltaic (PV) (Section 2.2) and battery (Section 2.3). Next is the performance simulation model, which simulates the long-term system operation with an hourly time step; and an economic simulation model, which simulates the cash flow for the system's lifetime.

### 2.2 Solar PV model

#### 2.2.1 Model of the in-plane solar irradiance

The global horizontal irradiance (GHI) solar radiation data, is the irradiance falling on a horizontal surface, and PV arrays are usually installed inclined to the horizontal plane, this inclination needs to be accounted for when calculating solar irradiance incident on the PV array. Many models of varying complexity have been developed for calculating incident solar irradiance. In this study, the inclined-plane irradiance is estimated using the model of Muneer [56,57]. In addition, we used the model of Martin and Ruiz [58] to account for the increased reflectance at the module surface when the light arrives at an oblique angle to the module surface. Together, these two models yield *effective* irradiance i.e. the solar irradiance arriving at the PV cells in the module.

#### 2.2.2 Model of the PV power output

The power produced by the PV array is calculated as a function of in-plane solar irradiance falling on the PV array, which is calculated in the previous section. We apply the model described in Ref. [50], which describes the PV power as a function of in-plane irradiance and module temperature. For other assumptions about effects influencing PV power, see Ref. [25]. These models yield the DC power of the PV array, assuming maximum power point tracking and optimal module inclination and orientation.

### 2.3 Battery model

The *State of Charge* of a battery, SOC, is a useful parameter to calculate the battery performance. SOC denotes the present charge in the battery as a percentage of the total battery capacity. When a battery is charged or discharged, the voltage across the terminals of the battery *V*_*b*_ is a function of the battery current *(I*_*b*_) and the SOC:

(1) $$V_{b}=V_{b}\left(I_{b},SOC\right)$$

The dependence of *V*_*b*_ on *I*_*b*_ and SOC has been measured for several values of SOC and *I*_*b*_. (Refer to Ref. [25] for details of the measurements). The function *V*_*b*_ is then defined as a matrix of discrete values. Using these values, we can then find the value of *V*_*b*_ for any combination of values SOC and *I*_*b*_ by interpolation (or in some cases extrapolation). In the present study, lead-acid and lithium-ion batteries have been considered, using the data described in Ref. [25].

### 2.4 Load profile for each community

The electricity consumption profile is one important addition to this model. The model incorporates load profiles per population cluster created at 100 m^2^ resolution. The load profiles are produced using a bottom-up stochastic load profile simulation model, RAMP [59], with a methodology for load formulation and assignation to population clusters that expands on the one developed for M-LED in Ref. [60].

#### 2.4.1 Archetypes formulation

A set of 100 residential user archetypes is developed to compute the same number of load curves [61]. The user archetypes are computed based on a variation of 3 parameters: Latitude (5 areas), Climate Zone (4 Zones), and Socio-Economic Conditions (5 Levels) of the user. Based on the variation of such parameters, the different users are characterized by different baskets of appliance ownership and patterns of use during the day. The obtained archetypes of users in terms of appliance ownership and use are fed in RAMP resulting in 100 different specific load curves.

#### 2.4.2 Parsing

To parse the heterogeneous RAMP residential load profiles to households in each population cluster, we expand the M-LED methodology developed in Ref. [60] and propose a novel, generalizable methodology that we showcase for the case of Nigeria. Specifically, using the cluster-level Relative Wealth index (RWI) [62,63], we define five quintiles of wealth and assign each population cluster to one of them. We also extract the prevalent urbanisation type of each cluster based on the GHS SMOD degrees of urbanisation classification [64] to determine the average number of people per household in each cluster based on the Nigeria Living Standards Survey 2020 rural-urban average household size statistics [65]. Based on such information and the population count in each cluster, we derive the number of households in each cluster.

Subsequently, we define four major climate types and assign each cluster to one of them based on the yearly number and seasonal distribution of Cooling Degree Days (CDD), a typical metric for determining cumulative heat exposure. We use CDDs based on a threshold temperature of 26 °C (CDD26) based on 1970–2000 monthly average data from WorldClim [66]. The four climate types (defined to capture heat exposure and seasonal variation) are defined as follows.

1. Climates where year-wise monthly mean CDD26 > 90
2. Climates monthly mean CDD26 > 90 between April and September and monthly mean CDD26 < 90 in the other months
3. Climates where monthly mean CDD26 < 90 between April and September and monthly mean CDD26 > 90 in the other months
4. Climates where year-wise monthly mean CDD26 < 90

We also define five latitude zones (to capture lighting requirements seasonality) based on distance from the equator.

• Zone 1: between 10 and 20° North of the Equator
• Zone 2: between 10° North or South of the Equator
• Zone 3: between 10 and 20° South of the Equator
• Zone 4: between 20 and 30° South of the Equator
• Zone 5: more than 30° South of the Equator

Based on the RWI quintile, climate zone, and latitude zone, we then parse the RAMP load profiles.

## 3 Data

### 3.1 Input data for determining the load profile for each community

Table 2 provides the references of the datasets used in generating the population clusters and load profiles per cluster

**Table 2:** Data sources for creating the load profiles for the population clusters.

Data	Year	Description	Reference
GHSL Population	2019	Global Human Settlement Layer at 250 m resolution	[67]
GHSL SMOD	2019	Global Human Settlement Layer at 250 m resolution	[67]
Worldpop	2020	Population counts of the constrained population for Nigeria (100 m resolution)	[68]
Worldpop	2020	Population counts of the non-constrained population for Nigeria (100 m resolution)	[69]
Worldpop	2020	Counts of buildings per grid cell (100 m resolution)	[70]
Relative wealth index	2022	The Relative Wealth Index predicts the relative standard of living within countries	[71]
Country boundary	2020	EUROSTAT 2020	[72]
Gridlines	2020	The existing and planned electricity grid in Africa	[73]

### 3.2 Weather resources data

Solar radiation data have been obtained from satellite-based algorithms [[74], [75], [76]]. The data are available as the SARAH Climate Data Record (PVGIS-SARAH2) with a temporal range from 2005 to 2020 produced by CM SAF [77]. The solar radiation data include both the global horizontal and direct horizontal irradiance; this makes it possible to calculate the irradiance on inclined planes, which is the typical configuration for PV modules. The time resolution of the solar radiation data is hourly, and the spatial resolution is 3 arc-minutes (about 5 km). Note that this resolution is finer than the resolution used for the calculations used in this study (6 arc-minutes).

Air temperature (at 2 m above ground) is used for modelling the instantaneous PV output power and to calculate the local air density. For this study, we have used 2 m temperature data from the new ECMWF ERA5 reanalysis. These data are available at hourly time resolution with a spatial resolution of approximately 31 km worldwide. At the time of writing, the period available is 2010–2017, which should be extended to 1979 - present during 2018. The relatively coarse resolution could lead to errors in mountainous areas with large variations in elevation or near the coastlines. At the time of writing ECMWF ERA5 data can be accessed from Ref. [78].

### 3.3 Technical and costs parameters of components

#### 3.3.1 Technical and costs parameters of PV module

The solar PV module assumed in this study monocrystalline PV module. The CAPEX for a solar installation (without battery storage) in Sub-Saharan Africa can vary widely between 0.6 and 1.6 EUR/Wp [79]. Considering these ranges, the capital cost is assumed at 830 EUR/kWp. The operation and maintenance cost (O&M) is assumed at 5 EUR/kW/year, which is mainly for module cleaning and repairs. The detailed technical parameters are summarized in Table 3.

**Table 3:** Technical and cost parameters of PV module.

Category	Parameter	Value	Units
Technical parameters	Nominal efficiency	20.30	%
	Max. power voltage/current	39.8/9.29	Vdc/Adc
	Lifetime	25	year
Cost parameters	Capital cost	830	EUR/kWp
	O&M cost	5	EUR/kWp/year

#### 3.3.2 Technical and costs parameters of the battery

There are various battery technologies and chemistries, with different technical parameters and characteristics. The lithium-ion battery is assumed in this design. The summary of the major parameters of the battery is tabulated in Table 4. The parameters are obtained from the International Renewable Energy Agency database [80].

**Table 4:** Technical and cost parameters of the battery.

Category	Parameter	For mini-grid	For standalone	Units
Technical parameters	Chemistry	NCA	LFP	–
	Round trip efficiency	95	92	%
	Depth of discharge	90	90	%
	Lifetime (throughput)	2500	2500	kWh/1 kWh
	Lifetime (calendar)	12	12	year
Cost parameters	Capital cost	352	578	EUR/kWh
	O&M cost	6	6	EUR/kWh/year
	Replacement cost	145	224	EUR/kWh

LFP: Lithium iron phosphate; NCA: Nickel cobalt aluminium oxide.

### 3.4 Key incentives considered

Incentives are crucial for private sector participation in providing electricity access in most of the rural areas in Sub-Saharan African countries. These incentives play a pivotal role in bridging the financial gap between the expenses associated with reaching remote areas and the ability to pay of these customers to cover upfront installation and operating costs [45]. Acknowledging this crucial link, governments and development partners are actively formulating diverse support packages to incentivise private sector investments [81]. This study focuses on three existing key incentive policies in Nigeria, investigating their potential impacts on electrification options within the country.

#### 3.4.1 Concessionary loans

Concessional loans are characterized by more favourable terms than what borrowers could secure in the open market. The concessional terms may include low interest rates, long tenors, or a combination of both. In Nigeria, commercial bank loans typically carry interest rate ranging from 18 to 22% with a tenor of fewer than 5 years. The subsequent list outlines some of the existing concessional loans available in Nigeria.

• Solar Power *Naija* (SPN) – SPN was designed to expand electricity access to 25 million individuals with five million new connections through the provision of stand-alone systems or a mini-grid. The initiative is a 140 billion Nigerian Naira (328 million EUR) long-term, 9% interest rate credit facility for private sector developers [82].
• Bank of Industry's Solar Energy Fund (SEF) – SEF is a 6 billion Nigerian Naira (14.1 million EUR) facility to enable commercial and rural households to acquire reliable solar solutions using a long-term loan at 9% interest [83].
• Sustainable Use of Natural Resources and Energy Finance (SUNREF) – SUNREF Nigeria project seeks to improve access to energy through improved access to affordable finance for renewable energy and energy efficiency technologies. The facility offers the private sector competitive loans and technical assistance for structuring green investments. The facility is a low-interest and long-term loan (8% interest) [84].

#### 3.4.2 Capital-based incentives

There are various capital-based incentives in Nigeria for renewable energy deployment both for mini-grid and SASs. Some of these programs include:

• Performance-based grant program (PBG) – This program aims to develop mini-grids on a rolling basis [85]). The communities are identified, verified, and sensitized by mini-grid private developers. Grants of approximately 600 EUR/connection are given, with a minimum total grant request of 10,000 EUR per mini-grid. Eligible projects are solar and solar hybrid systems in unserved areas, with a generation capacity of not more than 1 MW. The total funding available is 150 million EUR.
• Minimum Subsidy Tender (MST) – MST aims to kick-start the Nigerian market and catalyse mini-grid deployment at scale [86]. Mini-grid private developers will compete based on quality (technical proposal) and price (minimum subsidy requirement) to build, own, and operate solar hybrid mini-grids.

#### 3.4.3 Financing equipment for productive use

According to multiple studies [87,88], socio-economic development in remote communities is not guaranteed after gaining electricity access. Productive use of electricity needs to be an integral part of electrification planning. There are various facilities in Nigeria that provide funds for the productive use equipment in the form of grants or concessional loans, some of these facilities are:

• Result-based financing for productive appliances and equipment – Nigerian Rural Electrification Agency has set aside 19 million EUR to finance equipment for productive use [89] in remote communities.
• Energising Agricultural Programme (EAP) – This is a 3-year facility jointly developed by REA and RMI and funded by the Global Energy Alliance for People and Planet (GEAPP). The programme is a market-led solution that increases off-grid electrification while driving agricultural development. The payback period of most productive use equipment is under 12 months [45].

### 3.5 Policy incentives: input parameters

Table 5 presents the range of parameters of the analysed policies in Nigeria and the parameters selected for each of the policies analysed. The base case parameters correspond to the scenario where no incentives are applied. This is not a realistic scenario as there are many existing incentive policies; however, the scenario provides the basis of the current Nigerian market and therefore can be a reference for comparison.

## 4 Results and discussion

This section presents the outcomes of our analysis of the impact of incentives on the optimal electrification option for Nigeria. We evaluated the effectiveness of three incentive policies: concessionary loans, capital subsidies and financing equipment for productive use. The least-cost electrification option was calculated for each of the 22,696 population clusters in Nigeria, aggregating 129 million people. The least-cost option was calculated for the 4 scenarios. Additionally, the optimised system size and economic parameters of the systems, such as LCOE, CAPEX, and OPEX were determined. The three electrification options considered were grid extension, renewable energy mini-grid, and SASs. The configurations for renewable energy mini-grids and SASs included the integration of energy storage systems (ESS) combined with PV. The following section presents the effects of the three incentive policies on both the electrification options and economic aspects of the systems, accompanied by a sensitivity analysis for each incentive policy.

### 4.1 Effect of incentives on electrification options

Under the baseline scenario, the absence of incentives renders mini-grids and SASs less attractive from an investment perspective. Grid extension accounts for approximately 66%, while mini-grids and SASs account for 27% and 7% respectively. The relatively high percentage of grid extension is because, in Nigeria, communities have a shorter average distance from the existing grid and relatively higher population density in comparison with other Sub-Saharan African countries. Although this scenario does not reflect the current situation, as several incentive policies are already in place, it provides a useful reference for evaluating the impacts of the three incentive policies.

The concessionary loan scenario is simulated with a discount rate of 9%, the interest rate used by most concessionary loans to finance renewable energy projects in Nigeria, such as the Bank of Industry solar loan [83] and SUNREF facility [84]. The results reveal that both mini-grids and SASs become more attractive investment options. The share of mini-grids increases from 27% to 37%, a 10% increase, while the share of SASs rises from 7% to 12%, a 5% increase. Low-interest loans effectively decrease the LCOE of both mini-grid and SAS, making them more competitive against grid extension.

Assuming a capital subsidy of 600 EUR rebate per connection (household) for mini-grids and solar stand-alone systems in unserved and underserved communities, as currently provided by the Nigerian Rural Electrification Agency, we found that the share of mini-grid and SASs in the electrification options increases. Mini-grids experienced the largest increase, from 27% in the baseline case to 41%, whereas SAS rose from 7% to 12%. Our analysis indicates that both concessional loans and capital subsidies have the same impact on SAS, with both incentives increasing its share to 12%. However, this is not the case for mini-grids, where capital subsidy proves more effective in lowering the LCOE, boosting its share to 41%, in contrast to 37% for concessional loans.

Granting a loan or grant to acquire equipment for productive use in each community, as part of the financing equipment for productive use incentive, leads to an increase in daytime electricity demand due to the operation of acquired equipment for productive purposes (the equipment and power rating assumptions are included in the Appendix). As a result, more favourable conditions for optimizing PV systems emerge, leading to an increase in the share of SASs and mini-grids from 7% to 15% and 27% to 43%, respectively.

**Fig. 2:**
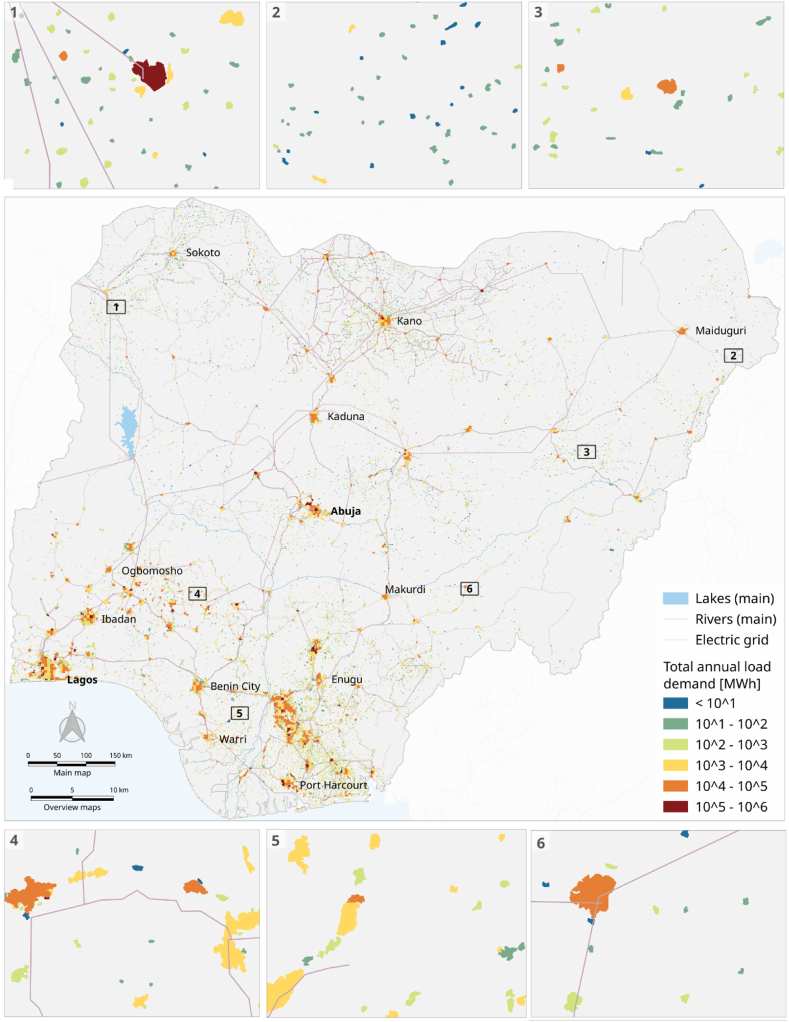
Total annual load demand (MWh) per population cluster at 100 m^2^ spatial resolution.

**Fig. 3:**
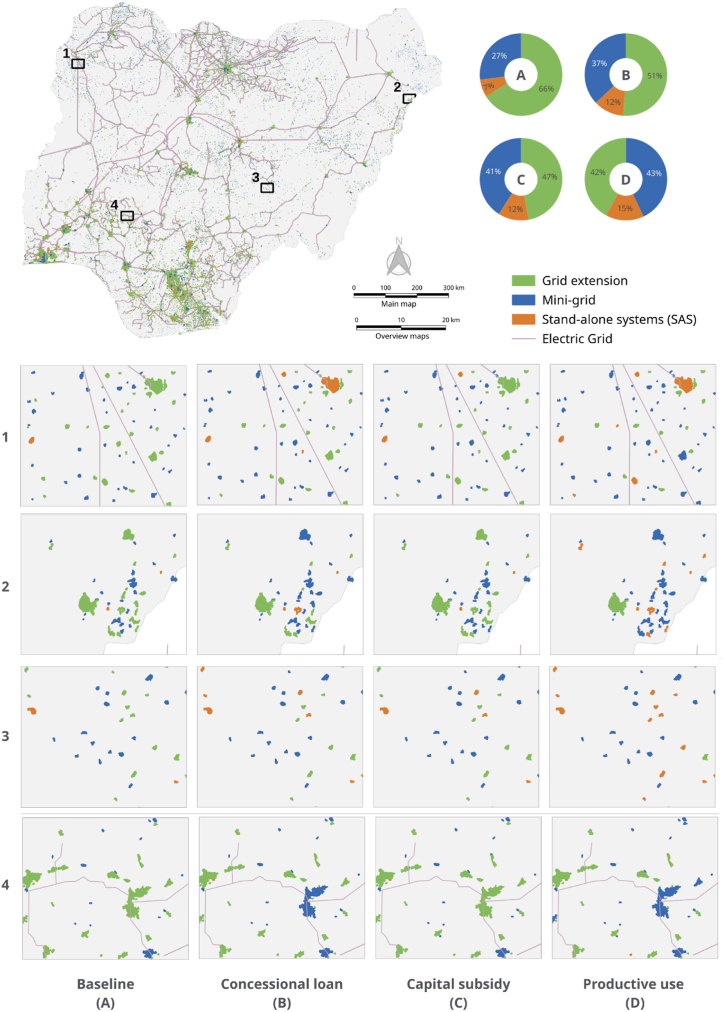
Comparison of the electrification options under the baseline and the three incentive policies. **(A)** Baseline**, (B)** Concessional loan. **(C)** Capital subsidy **(D)** Financing productive use equipment.

Fig. 3 illustrates the three possible electrification options under the baseline and three incentive policies. We selected four areas (marked 1, 2, 3, and 4 on the map) to display the transformation of electrification options before and after applying various incentive policies for the clusters under different geographical, demographic, and economic conditions (also refer to Fig. 2 for total load demand per cluster) and infrastructure conditions (i.e. existence of grid). The map clearly shows that incentive policies can modify the electrification option of the baseline scenario.

Consider the example of the clusters depicted in Fig. 3 within square 3. In the baseline scenario, the electrification option for the largest cluster in this area is grid extension. However, upon applying a concessional loan incentive, the cluster shifts to a mini-grid. Next, when a capital subsidy is introduced the cluster reverts to grid extension. Finally, with the implementation of a productive use incentive, the cluster switches once again to a mini-grid.

### 4.2 Variation in economic parameters resulting from the incentive policy applied

**Fig. 4:**
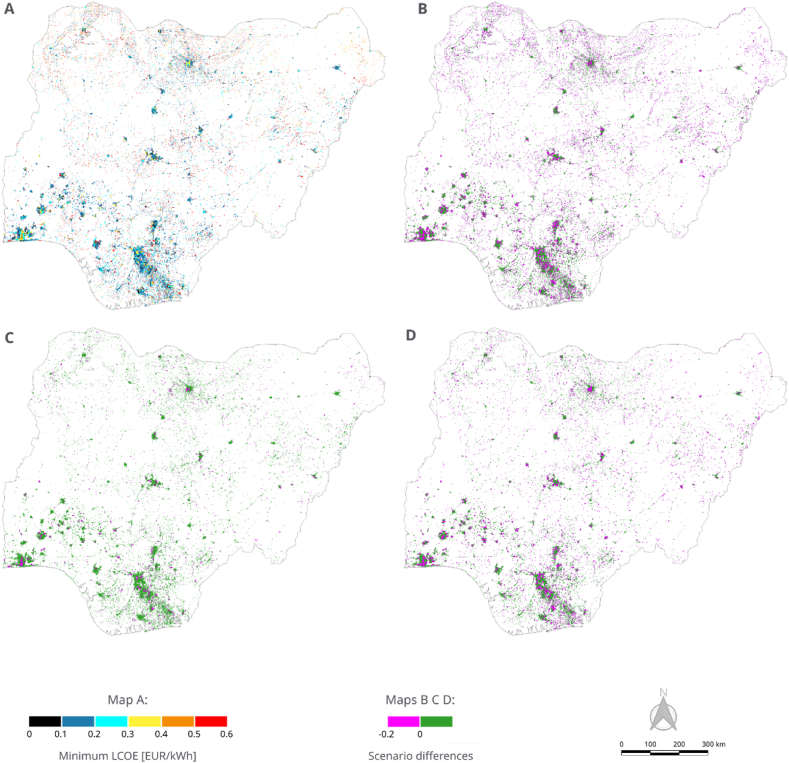
(A) LCOE of the least-cost electrification options for the baseline case. **(B)** Differences between the LCOE in the baseline and the LCOE under the concessionary loans. **(C)** Differences between the LCOE in the baseline and the LCOE under the capital subsidy case. **(D)** Differences between the LCOE in the baseline and the LCOE under the financing productive use equipment case.

The implementation of incentive policies affects the LCOE, CAPEX, and OPEX of the least-cost technology options, as illustrated in Fig. 4. In the baseline scenario, the average LCOE is 0.34 EUR/kWh. Following the application of incentive policies, the average LCOE decreases to 0.31 EUR/kWh for concessionary loans (Fig. 4. B), 0.30 EUR/kWh for capital subsidy (Fig. 4. C), and 0.27 EUR/kWh for financing productive use (Fig. 4. D).

**Fig. 5:**
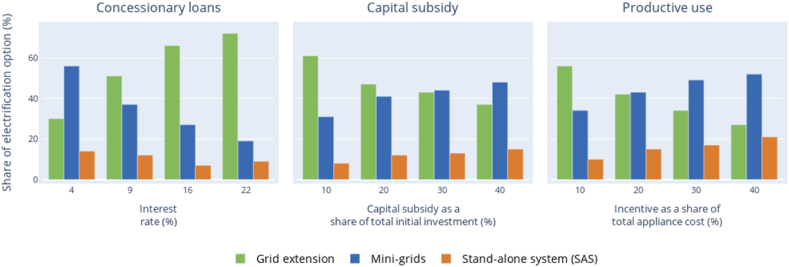
The impacts of variation of various incentive policies on the least-cost electrification option.

Fig. 4 (A) displays the LCOE distribution across all 22,696 computed clusters, ranging from 0.13 to 0.56 EUR/kWh. In Fig. 4 (B, C, D), the green shading represents clusters exhibiting no variation in LCOE when incentives are applied, while pink highlights clusters where incentives result in a reduction of up to 20 cents in the average LCOE. Notably, both concessionary loans and financing for productive use contribute to a similar decrease in LCOE within these clusters; however, the impact of concessionary loans on reducing the average LCOE is more pronounced.

### 4.3 Sensitivity analysis on the effects of incentive policies

**Fig. 6:**
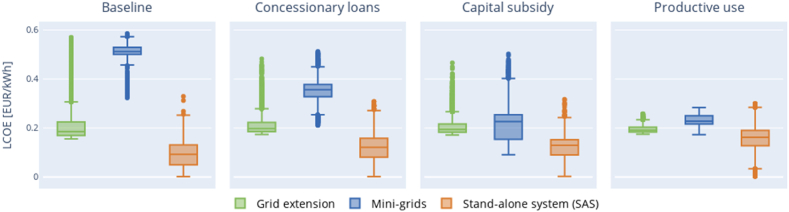
The impacts of variation of incentive policies on the LCOE.

The policies simulated in the preceding sections are the typical policies currently available in Nigeria. However, these policies vary widely in terms of both amount and scope. Understanding how their variation will affect the electrification options will inform policymakers in formulating new and streamline existing policies. Therefore, we perform sensitivity analysis to understand how the variation affects both the least-cost electrification option (Fig. 5) and LCOE (Fig. 6).

**Table 5:** Parameters considered without incentive policies (base case), range of values per each analysed policy and selected value.

Policy	Without incentives (Base case)	Value selected for each incentive	Range of existing policies
***Capital subsidy***			
Rebate (EUR/household)	None	350	100–600
***Concessionary loan***			
Interest rate (%)	15.42	9	6–12
Loan term (years)	5	8	7–12
Loan percent (% of capital cost)	0	100	50–100
***Financing equipment for productive use***			
Cost of productive use equipment (% of capital cost)	None	30	20–50
Demand increase as a result of productive use equipment	None	2 kWh demand increase for every 200 EUR	Depends on the percentage of capital cost

The concessionary loan variation reveals a consistent impact on the proportion of mini-grids and SASs (Fig. 6 a). The variation in the interest rates from the base case (16%) results in consistent changes in the shares of mini-grids and SASs. The lowering interest rates increase the mini-grid and SAS shares, in case of rate spikes the mini-grid shares decrease. The mini-grid option requires higher up-front but lower O&M costs compared to the flattened grid electricity monthly tariff. The higher interest rates weigh the future downpayments less, so it favours grid-based electricity. This financial preference pattern is also similar for capital subsidy and financing productive equipment (Fig. 6 b-c). However, the effect is not uniform for mini-grid and SASs. The increase/or decrease of the incentive policies has more impact on the mini-grid than the stand-alone system. This can be explained because, per capita, mini-grids have higher capital costs in the upfront period because of the distribution network, than for SASs, for the same community. The implication of this is that the incentive type and amount should be varied across technology options to be more effective. This is not the case, at least for Nigeria, where an equal amount of incentive is given for both mini-grid and SASs. This uniformity has a direct consequence that applying the same incentives favours the SASs over the mini-grid options, however in some cases this will lead to undersupply of electricity where higher demand productive or public uses (education or health facility) would require mini-grids instead of SASs.

Fig. 6 analyses the results of the sensitivity analysis of the LCOE for each of the technologies under the 4 scenarios, independently if they are the least cost option. For comparison, the green column shows the least-cost LCOE distribution for each of the scenarios.

## 5 Conclusion and policy implications

### 5.1 Conclusions

Our study demonstrates that carefully designed incentive policies can significantly affect the optimal electrification strategy in Nigeria, steering the country toward more sustainable and cost-effective solutions. By evaluating the efficacy of different incentives, policymakers can optimize their strategies to achieve universal access to reliable, affordable, and clean energy. In this study, we specifically analysed the impacts of three prevailing incentive policies in Nigeria on the optimal electrification options (grid extension, mini-grids, and SASs) and the associated economic parameters. The least-cost electrification model was applied to 22,696 population clusters in Nigeria. The results indicate that the incentive policies affect the electrification options at variable magnitudes. Under the baseline case, the optimal electrification option for the majority of the population clusters is grid extension at 66%, followed by mini-grid at 27%, and SASs at only 7%.

Upon the application of incentives, there is an increase in the share of both mini-grids and SASs. The change in the optimal electrification portfolio shares is influenced by the different impacts of the support policy and their implied discount rates on the resulting electricity generation costs for grid extension, mini-grid, and SAS options. As the different support policies have distinctive impacts on the technologies (due to the different cost structures; in up-front and O&M components) and on locations (attributed to the different irradiation, loads, and settlement types), our presented methodology can help policymakers identify optimal incentive schemes for specific regions and communities. Some of the most pronounced impact pathways identified include the application of concessionary loans having a stronger impact in increasing the share of mini-grids compared to SASs; the subsidies in capital investments increasing the share of both mini-grids and SASs, with a more pronounced effect on mini-grids; financing appliances for productive use leading to an increased share of both mini-grids and SAS compared to the other incentive policies. This last impact is particularly important, as mini-grids with higher electricity capacities than SASs are better suited for the higher demand profiles required by productive use.

### 5.2 Innovations and limitations

The present study builds upon the initial model proposed by Huld et al. (2017) [25] by incorporating methodologies from Husein et al. [54] to enhance its capabilities. The enhanced model includes the integration of various renewable energy technologies, expansion to urban and suburban areas, and the inclusion of grid extension as an electrification option. Notably, the novelty in this model is that the demand side now utilizes specific hourly load profiles for each population cluster, considering the socio-economic heterogeneity of the population and geography and climate-related factors. Additionally, another notable improvement is that the model performs techno-economic analyses with variable parameters, conducting a detailed hourly simulation of system performance, leading to a more accurate sizing of system components and LCOE computation, albeit at a higher computational cost.

Despite these advancements, our study has limitations that provide opportunities for further research. Firstly, the selection criteria for the best electrification option are based solely on cost and reliability, without explicit consideration for emission reduction and climate change mitigation. Secondly, this study assumes sufficient generation and transmission capacity for grid extension, which is not the case in Nigeria. Therefore, the additional investment required for augmenting generation and transmission capacity has not been factored into the study.

These limitations notwithstanding, the present study offers valuable insights into the types of incentives that could be offered to promote the adoption of optimal electrification solutions for different clusters of communities. Moreover, the study highlights the importance of considering various factors, including socio-economic heterogeneity, geography, and climate-related factors, when evaluating electrification options. Future studies can build upon these findings to develop even more comprehensive models that address the limitations mentioned above and provide more holistic recommendations for electrification strategies.

### 5.3 Policy implications

The present study carries significant policy implications. The findings indicate that different incentive policies yield dissimilar effects on mini-grid and SASs deployments. Specifically, the results suggest that capital subsidy may be more effective in reducing the mini-grid cost in certain communities, whereas the reverse holds for SASs in other population clusters. Thus, policymakers should first use studies like this as a tool for informed policy development.

Moreover, the study reveals another policy implication, that for all the incentives considered, financing productive use equipment leads to a greater proportion of population clusters transitioning from SASs to mini-grids. This is a positive outcome since mini-grids are more sustainable and easier to scale up than SASs, with all the benefits of socio-economic development. Furthermore, policymakers can target areas where incentives would be most effective in promoting electrification, given the diversity of least-cost electrification alternatives across regions and communities.

Furthermore, the study implies that implementing incentive mechanisms resulting in lower LCOE would substantially diminish the optimal extent of grid extensions. This reduction in grid extensions could alleviate the public burden associated with state-owned companies executing these extensions, partly offsetting the costs of maintaining incentive programs. Therefore, national electricity network planning including regional master plans, should take this factor into account. The optimality maps generated by this study could be readily integrated into updates of these master plans. Finally, financing schemes for the appliances for productive uses would also positively influence local production, yielding additional economic benefits in terms of employment and trade.

## CRediT authorship contribution statement

**Munir Husein:** Writing – review & editing, Writing – original draft, Methodology, Investigation, Formal analysis, Data curation, Conceptualization. **Magda Moner-Girona:** Writing – review & editing, Writing – original draft, Supervision, Methodology, Investigation, Formal analysis, Data curation, Conceptualization. **Giacomo Falchetta:** Writing – review & editing, Methodology, Formal analysis, Data curation. **Nicolò Stevanato:** Writing – review & editing, Methodology, Formal analysis, Data curation. **Fernando Fahl:** Visualization, Formal analysis, Data curation. **Sandor Szabó:** Writing – review & editing, Formal analysis.

## Declaration of competing interest

The authors declare that they have no known competing financial interests or personal relationships that could have appeared to influence the work reported in this paper.

## Figures and Tables

**Table A1:** Grid extension costs in Off-PVGIS model

Parameter	Value	Unit
Lifetime	30	Years
MV line cost	9000	EUR/km
Transformer	5000	EUR/50 kVA
Connection charge per household	125	EUR/Household
O&M costs of distribution	2	% of capital cost per year

**Table A2:** Power requirements, costs, and indicative payback periods of selected income-generating appliances

Sector	Activities/Appliances	Power required	Cost from supplier (EUR)	Payback period (months)
Primary industries (Agriculture, fishing)	Egg incubator	80–160 W	50 to 100	1 to 3
	Grinder for pulse and beans	5.2 kW	1500 to 4000	6 to 12
	Water irrigation pump	3.7–22.4 kW	200 to 1000	3 to 6
	Sterilizer (for dairy processing)	3–6 kW	600 to 2000	1 to 3
	Packager	250 W to 3 kW	500 to 1000	6 to 12
Light manufacturing	Electronic welding machine	3–7.5 kW	200 to 300	6 to 12
	Jigsaw	400 W	100	3 to 6
	Electric drilling machine	400 W	20 to 50	3 to 6
	Popcorn maker	1.5–2.1 kW	50	1 to 3
Commercial and retail activities	Computer	15–100 W	250 to 800	3 to 6
	Printer/scanner for stationery	0.5–2 kW	150 to 250	3 to 6
	Sewing machine	200 W	30 to 100	3 to 6
	Television for local cinemas and bars (including decoder)	50–200 W	100 to 200	1 to 3
Total average		33.98 kW	6850	

**Table A3:** Steps of the clustering algorithm.

Step	Description
01:	Import the input data to GRASS GIS
02:	Calculate the population density maps (population/built-up areas) for both the constrained and non-constrained data
03:	Split the population density map into multiple layers based on the following criteria: a) Constrained: 100 inhab./km^2^ interval b) Non-constrained: two intervals only ( ≥ 1500 inhab./km^2^; ≥ 300 and < 1500 inhab./km^2^)
04:	Cluster each density interval layer using the following parameters: a) Constrained: first layer ( ≥ 1500): threshold = 0.01; min size = 300; other layers: threshold = 0.001; min size = 300 b) Non-constrained: first layer ( ≥ 1500): threshold = 0.01; min size = 300; other layers: threshold = 0.1; min size = 100
05:	Merge the cluster layers (constrained and non-constrained)
06:	Convert the raster to vector (polygons)
07:	Calculate the stats for each polygon (area, density, built-up share, and population) based on constrained population and density layers
08:	Split the vector layer in 4 classes, according to the following criteria (adapted from the GHSL SMOD methodology). class 1= (average density ≥ 1500 OR built-up share ≥ 0.5) AND total population ≥ 50000" class 2=(average density ≥ 1500 AND built-up share ≥ 0.5) AND POP_sum ≥ total population AND class is null class 3 = "average density ≥ 300 AND built-up share ≥ 0.03 AND POP_sum ≥ total population AND class is null class 4 = "average density ≥ 300 AND built-up share ≥ 500 AND POP_sum < total population AND class is null
09:	Remove small cluster areas ( ≤ 0.1)
10:	Merge the 4 classes layers into a unique vector layer
11:	Split large clusters (>20 km^2^) into small parts using a grid of 0:02:50 (around 28 km^2^)
12:	Calculate the final stats using the population, density, health facilities, and grid lines.
13:	Extract the centroids of each cluster and convert to csv file

**Table A4:** Cluster attributes.

Attribute	Description
cat:	unique ID
class:	cluster class based on criteria defined in Step 04 above
area_km^2^:	cluster area in km^2^
split:	large clusters split in small parts (split = 1) or original cluster areas (split = 0)
POP_minimum:	min population inside the cluster
POP_maximum:	the max population inside the cluster
POP_average:	mean population inside the cluster
POP_sum:	the total population inside the cluster
DEN_minimum:	min population density inside the cluster (inhab./km^2^)
DEN_maximum:	max population density inside the cluster (inhab./km^2^)
DEN_average:	mean population density inside the cluster (inhab./km^2^)
health_count:	health facilities inside the cluster (all facilities)
health_first:	health facilities inside the cluster (type first)
health_primary:	health facilities inside the cluster (type primary)
health_secondary:	health facilities inside the cluster (type secondary)
health_tertiary:	health facilities inside the cluster (type tertiary)
x:	longitude of the centroid
y:	latitude of the centroid
distance_grid_km:	distance from the centroid to the nearest grid line (km)
